# An early intervention to promote maternal sensitivity in the perinatal period for women with psychosocial vulnerabilities: study protocol of a randomized controlled trial

**DOI:** 10.1186/s40359-020-00407-3

**Published:** 2020-04-28

**Authors:** Anne Kristine Aarestrup, Mette Skovgaard Væver, Janne Petersen, Katrine Røhder, Michaela Schiøtz

**Affiliations:** 1 Center for Clinical Research and Prevention, Frederiksberg Hospital, Nordre Fasanvej 57, Vej 8, Indgang 1, 1.sal, 2000 Frederiksberg, Denmark; 2 Centre for Early Intervention and Family Studies, Department of Psychology, University of Copenhagen, Copenhagen, Denmark; 3 Section of Biostatistics, Department of Public Health, University of Copenhagen, Copenhagen, Denmark

**Keywords:** Early intervention, Maternal sensitivity, Parenting education, Preventive intervention, Prenatal screening

## Abstract

**Background:**

Maternal mental well-being and social circumstances during pregnancy and early childhood impact the child’s well-being and development. Supportive and sensitive parenting is one of the strongest predictors of positive emotional, social and behavioral outcomes for the child. Knowledge is needed about how to detect and support vulnerable families already during pregnancy and in the postnatal period. The aim of this study is to assemble and evaluate an interdisciplinary cross-sectoral intervention to promote maternal sensitivity among women with psychological or social vulnerabilities.

**Methods/design:**

This randomized controlled trial tests the efficacy of an intervention program in the perinatal period compared to care as usual in enhancing maternal sensitivity in a group of psychologically or socially vulnerable women in the Capital Region of Denmark. The intervention consists of four components: 1) detecting symptoms of mental illness in vulnerable pregnant women and initiating treatment if indicated, 2) strengthening parenting skills using the Circle of Security Parenting program, 3) supporting breastfeeding, and 4) sharing knowledge and organizing treatment pathways for families across sectors. Seventy-six families will be randomly assigned to the intervention or care-as-usual. Data will be obtained at study inclusion at 3–5 months of pregnancy, eight weeks after childbirth, and nine months after childbirth. The primary outcome is maternal sensitivity. Secondary outcomes include infant’s socio-emotional development, parents’ mentalization, parental stress, depressive symptoms, and parental wellbeing. Qualitative data will provide insight into the implementation process.

**Discussion:**

This paper presents the rational and background for developing the intervention. Furthermore, the design and protocol of the randomized controlled trial. It is hypothesized that the intervention will be associated with positive changes in primary and secondary outcomes. If effective, the intervention will provide insights into prenatal risk profiles among an identified group of psychosocial vulnerable women important for early screening and point to effective preventive interventions that can support women in the perinatal period, increase maternal sensitivity and promote positive child development -starting before the child is born.

**Trial registration:**

ClinicalTrials.gov: ID: NCT03190707. Registered June 16, 2017.

## Background

There are disparities between social groups in health and in access to public health services [1]. Social inequality in health is established as early as before birth and continues throughout life. Development in utero forms the foundation for physical, mental, and cognitive capabilities and health status later in life [2], and fetal development is affected by the mother’s behavior and wellbeing [3]. After birth, supportive and sensitive parenting is one of the strongest predictors of positive developmental outcomes for children. Longitudinal studies from different countries show that consistent sensitive and supportive parenting predicts lower levels of child problem behavior and enhanced cognitive development [4–9]. Conversely, harsh inconsistent parenting is predictive of poor child outcomes: child abuse and neglect, delinquency, drug use, school failure, poor health and mental health, and partner violence [10]. Parenting seems to be the mechanism or “common pathway” through which risk factors such as parent mental health problems, stress, family poverty, low education etc. impinge on children. Good parenting appears to be able to protect children in the face of these stresses [11, 12].

During infancy, an important developmental milestone is for the infant to form a secure attachment relationship with its parents. There is evidence that maternal sensitivity is causally related to positive child development [13–15]. Sensitive and responsive parenting is established as the most important predictor of secure attachment [16]. Sensitive parents are aware and capable of understanding their child’s expression of emotions and respond to the child’s need in a timely and appropriate manner. Conversely, lack of or inconsistent accessibility/presence or misunderstanding the child’s emotional expression and behavior can lead to an insecure or disorganized attachment relationship when frightening the child [17]. Secure attachment has been shown to be significantly associated with several positive outcomes for the child in terms of emotional, social, and behavioral development and adjustment, as well as school performance [18]. Likewise, insecure and disorganized attachment is associated with an increased risk for the development of externalizing and internalizing behavior [19, 20] and later psychopathology [21].

### The perinatal period

The mother-infant relationship starts developing during pregnancy [22].The mother’s representational and behavioral involvement with her fetus (e.g. the maternal-fetal relationship or prenatal attachment) is thought to help the mother in the psychological preparation in the transition to parenthood [23, 24]. Meta-analytic evidence supports the notion that the mother’s prenatal involvement with her developing child is predictive of the quality of maternal behavior after the birth as well as infant attachment classification [25, 26]. In addition, a good maternal-fetal relationship is associated with better prenatal maternal health behavior, such as eating healthy, exercising, attending prenatal assessments, and avoiding use of alcohol and drugs, thereby protecting fetal development [27].

#### Breastfeeding

It is also during pregnancy, that the decision to breastfeed is formed. Breastfeeding might be associated with increased maternal sensitivity. A large American study shows that breastfeeding is associated with increased maternal sensitivity towards the child and more appropriate and effective responses to the child, strengthening the quality of the mother-child relationship [28]. This finding is supported by a large Dutch study, that showed that duration of breastfeeding was associated with increased maternal sensitivity as well as more secure infant attachment and less disorganized infant attachment [29]. Also, Britton et al. (2006) concluded in their study that mothers who chose to breastfeed showed enhanced sensitivity during early infancy which may foster secure attachment [30].

Besides potential relational benefits breastfeeding also has numerous beneficial effects in terms of protection against childhood diseases [31, 32]. Young women and those with limited or no education, from a lower socioeconomic group or with an unstable employment history often quit breastfeeding earlier than other women [33]. A number of studies have shown that maternal self-esteem and intentions related to breastfeeding are of major importance [34]. In addition, women with knowledge about breastfeeding continue to breastfeed over a longer period of time; however, despite a generally high level of knowledge about breastfeeding, they may lack knowledge about how to succeed with breastfeeding [35]. The social network of the woman and/or the couple also impact the course of breastfeeding [36].

### Psychosocial vulnerability

The mother’s psychological health and social background are of crucial importance to her well-being and that of her child. Mental health problems are common during the perinatal period. Prevalence rates suggest that up to 15.6% of pregnant women and 19.8% of new mothers are affected by non-psychotic mental disorders with depression being most common (11 and 13% respectively) [37]. Perinatal depression has been associated with lower maternal-fetal involvement, lower maternal sensitivity, less secure attachment and more insecure attachment, and has been found to have long-term consequences for child development, including increased risk for emotional, behavioral and cognitive problems [38–41].

However, not all studies support these associations and it has been suggested that it is the combination of risk factors that puts the child at risk not the presence of depression per se [37, 42, 43]. It might be that persistent psychological difficulties, such as personality disorder or insecure attachment representations, also affects the mother’s risk status placing her at risk of non-optimal parenting and her child in developmental risk. Insecure attachment has in several studies been linked to risk of perinatal depression [44] and suggested as a moderator of the association between maternal depression and parenting [45]. Adaptation to motherhood can be particularly challenging for women with personality disorder since they have additional problems with interpersonal functioning, affective disorders and impulse control; the presence of personality disorder can adversely affect parenting [46]. A recent study demonstrated that postpartum depression only posed a risk factor for infant insecure attachment in the face of maternal comorbid personality disorder [42].

Preventive interventions during early childhood are more effective than those that occur later in a child’s life [2]. Pregnancy offers a unique period for buffering the effect of social inequality in health as interventions can be initiated before the child is born. Preventive parenting interventions presume adequate detection of at-risk future parents, effective perinatal interventions, and an organizational framework for the often intersectoral and interdisciplinary work that takes place during pregnancy and the early postnatal period.

It is crucial to identify at risk mothers during pregnancy and offer treatment to prevent the potential effect of maternal distress on the child’s wellbeing and development [47]. However, only treating maternal psychopathology does not necessarily lead to an improvement in the quality of parental behavior. This suggests that treatment of mental health problems should be accompanied by interventions that focus on supporting vulnerable mothers in enhancing a good relationship with their child [48, 49]. Findings from individual studies and systematic reviews show that interventions targeting parents can promote healthy relationships between parents and child and promote development and well-being for both child and parents [50–52]. One intervention program that appears to be effective at improving maternal sensitivity and infant attachment is an attachment-based parenting program - the Circle of Security Parenting Intervention (COS-P) [53, 54]. COS-P is a DVD-based parent training program targeting parents with children aged 0–5 years who are at risk of or have developed insecure attachment relationships with their caregivers. Through COS-P, parents learn how to recognize and interpret the child’s emotional needs and how to respond appropriately to support the child’s socio-emotional development and well-being. Parents or other caregivers receive the chapters individually or in groups offered by a therapist certified in COS-P [54].

In Denmark, pregnant women with complex psychosocial difficulties are referred to an extended interdisciplinary antenatal intervention that includes collaboration with different health professionals in the hospital sector or municipality [55]. Several group-based treatment programs for vulnerable pregnant women and families with infants already exist in Danish municipalities and regions. However, studies have shown that vulnerable groups may experience negative effects of engaging in group-based interventions [56], and several group-based interventions are challenged by dropout and lack of participation during meetings [57]. Consequently, despite a pressing need for health promotion and prevention, vulnerable pregnant couples often end up with treatment programs that are worse than those of pregnant couples who are not as socially disadvantaged as they do not participate in the programs being offered [58]. Furthermore, a new evaluation of existing services in the Danish Healthcare System reveals a need to strengthen the interdisciplinary and intersectoral collaboration between general practice, the hospital, and the municipalities focusing on communication, coordination, and joint interventions to prevent gap between pre- and postnatal services [59]. To our knowledge no previous studies have examined the effect of integration between pre- and postnatal care to vulnerable pregnant women. With this study, we aimed to develop and evaluate a new intersectoral and interdisciplinary perinatal intervention to women with identified psychosocial difficulties to strengthen maternal sensitivity already during pregnancy.

### Study aims and hypothesis

The overall aim of the study is to test the efficacy and effect of an early interdisciplinary, intersectoral and perinatal parenting intervention for at-risk pregnant women delivered by midwifes and health nurses. The hypothesis is that the intervention will promote maternal sensitivity in interactions with the infant nine months after the birth (primary outcome).

Secondary outcomes and hypothesis are that the intervention will lead to: Better infant socio-emotional development, better maternal ability to mentalize, reduced parental stress, reduced depressive symptoms, and improved maternal wellbeing.

Additional aims of the study:

1. Identifying psychological risk factors important for prenatal bonding among pregnant women at psychosocial risk with the long-term aim of strengthen prenatal screening programs. Our hypothesis is that attachment theoretical models improve our understanding of prenatal risk and resilience.
2. Identifying elements of importance for the delivery of integrated perinatal care across disciplines and health care sectors and investigating the vulnerable families’ experience of the interdisciplinary and intersectoral care package. We expect an improved collaboration between the two healthcare sectors and that families will experience this as positive.

## Methods

### Study design and setting

The project will be conducted as a randomized controlled trial. The intervention group will receive the intervention consisting of four components described below and the control group will receive care as usual. The study will take place in Herlev-Gentofte Hospital in Denmark and four of the affiliated municipalities: Ballerup, Gentofte, Herlev, and Rødovre. Intervention components will be delivered to families at the hospital during midwife consultations and in their homes by health nurses.

### Participants

The target population for the intervention in this study is women with psychological or social vulnerabilities. To be included in the study the women must be classified following official Danish Health Care recommendations [55] as having psychological or social vulnerabilities by the general practitioner and the hospital. Pregnant women should live in the municipalities of Ballerup, Gentofte, Herlev and Rødovre.

Exclusion criteria are: Unable to speak or understand Danish, under the age of 18, have a previous child who is placed in care outside the family during the intervention period, or at the time of inclusion have a registered or known maternal ICD-10 diagnosis of: Active eating disorder, severe depression, psychosis, schizophrenia, bipolar disorder, and severe obsessive compulsive disorder. Women with these diagnoses were excluded as they would be offered treatment in the existing psychiatric system.

### The intervention

The intervention consists of four key components initiated during pregnancy and continued postnatal until nine months after the birth of the child. The components are: 1) detecting symptoms of mental illness and initiating treatment if indicated, 2) initiating a health-nurse delivered, individual, attachment-based parenting program, 3) supporting breastfeeding decision and duration through education on techniques and strategies for successful breastfeeding, and 4) sharing knowledge and organizing treatment pathways for families across sectors to overcome a potential gap between pre- and postnatal care for pregnant and new mothers. The midwifes and health nurses are asked to register the intervention components provided to the intervention group in order to monitor how many components of the intervention families in the intervention group receive.

#### Detecting need for mental health treatment

Vulnerable pregnant women in the intervention group will receive an extended midwife consultation at the usual time of the second consultation at 20–22 weeks gestation. The midwife will screen the pregnant woman in the intervention group for symptoms of depression and risk for personality disorders. The approximately 20-min screening consists of the questions from the Edinburgh Postnatal Depression Scale (EPDS) [60] and the Standardized Assessment of Personality (SAPAS) [61] to screen for symptoms of personality disorder. Midwives will be trained in using the screening tools.

Pregnant women with EPDS scores ≥12 will be referred by the midwifes to a care package aimed at depression at the psychiatry unit in the Capital Region of Denmark during a monthly meeting with midwives and psychiatrist. If the pregnant woman answers question 10 about suicidal thoughts affirmative, the midwife will assess her current suicidal ideation and/or intent with subsequent action informed by clinical judgment by a psychiatrist. Women may be referred for examination at the psychiatric emergency room resulting in an explicit plan of action for midwives to follow. A SAPAS score ≥ 3 will similarly trigger a discussion of the participant during the monthly meeting between midwives and the adult psychiatry for the purpose of discussion referring to further treatment.

#### Strengthening parenting and maternal sensitivity

The COS-P intervention will be offered individually during home visits to pregnant women/mothers and their partners (when interested) in the intervention group by their health nurse. Vulnerable pregnant women in the intervention group will receive two additional home visits by the health visitor before birth and seven additional visits after birth. The visits will have a duration of 1.5 h and consist of eight chapters of the COS-P (Table 1).

**Table 1:** Overview of the COS-P visits

Visit	Time	COS-P module
1	Pregnancy week 32	Chapter 1 – Introduction to the Circle
2	Pregnancy week 34	Chapter 3 (first half of the module) – Managing your child’s emotions
3	Baby at 9 weeks	Chapter 4 – Organizing your child’s feelings
4	Baby at 11 weeks	Chapter 2 – Exploring your children’s needs
5	Baby at 13 weeks	Chapter 3 (second half of the module) - Managing your child’s emotions
6	Baby at 22 weeks	Chapter 5 – The path to security
7	Baby at 24 weeks	Chapter 6 – Exploring our struggles
8	Baby at 26 weeks	Chapter 7 – Rupture and repair in our relationships
9	Baby at 36 weeks	Chapter 8 – Summary and celebration

This is the first study to offer COS-P components during pregnancy. As the COS-P intervention has not been developed to be used during pregnancy where parenting – at least for primiparous mothers – are mostly based on expectations rather than experiences, the order of the COS-P sessions has been changed.

The chapters include the use of video material for identifying and addressing issues, such as attachment theory, regulating emotions and the parenting role. The video material presents examples of developmentally appropriate support for the child and problematic parent-child interactions. Parents will learn how to observe and interpret the child’s signals and to reflect on and verbalize strengths and weaknesses regarding their relationship and interactions with their child.

Health nurses will be certified in the use of COS-P during a four-day intensive training course. Adherence to the COS-P manual is assured by supervision to the health nurses from a COS-P supervisor.

#### Supporting breastfeeding

An additional intervention is educating the participating women and their partners about the importance of breastfeeding and how to do so successfully. This will take place during a one-hour joint consultation with the midwife and the health visitor focusing on techniques and strategies for successful breastfeeding. The teaching will use small videos and pictures of parents and babies in different situations to inform the parents of the importance of proximity and breastfeeding and to talk with the parents about their potential perceived challenges regarding breastfeeding. The teaching focus on four simple key messages; 1) skin-to-skin contact with the baby as often as possible and primarily with the mother 2) frequent breastfeeding when the baby desire it and at least eight times per day, 3) laid-back breastfeeding or another good position where the mother and the baby are in close physical contact and 4) breastfeeding is a common task for the parents. This consultation will take place at gestational week 28. Before the intervention begins, midwives and health visitors will receive oral and written introduction to the tasks related to the joint consultation from the project team.

#### Shared knowledge between sectors

Another aim of this joint consultation is the sharing of knowledge and the communication of information between the two sectors responsible for a shared plan for the care of the pregnant woman. This intervention component also includes systematic sharing of knowledge about the participant from the midwife at the maternity ward to the health visitor when she leaves the maternity ward. Before the intervention begins, midwives and health visitors receive oral and written introduction to tasks related to joint consultations and the systematic sharing of knowledge. This includes an instruction in how to structure the shared consultation with an introduction where the expecting parents introduce themselves and tells about the pregnancy, a following dialogue about the parents’ experiences, expectations and conceptions about parenthood, a dialogue about the challenges the expecting parents have and a dialogue about how the can get support from the healthcare system.

### Care as usual

Existing practice for pregnant women with complex psychosocial difficulties at Gentofte-Herlev Hospital and in the municipalities of Ballerup, Gentofte, Herlev and Rødovre will be offered to women in both the intervention group and the control group. In Denmark, perinatal care is guided by national guidelines from The Danish Health Authority [55]. An extensive level of universal support from general practitioners and midwifes during pregnancy and from health visitors to families with newborns during their first year of life is recommended. All pregnant women are offered a basic package of antenatal care consisting of three consultations with their general practitioner, two ultrasound examinations, and four-to-seven consultations with midwifes depending on parity [55]. Postnatal examinations of the infant are performed regularly by the health nurse in the infant’s home, including measuring growth and evaluating the infant’s physical and emotional development [55]. During the child’s first year, the health visitor examines the infant at least twice within the first three weeks after birth, at two months, at four months (for first-time mothers) and again at eight months. Extra counseling home visits to vulnerable families after the infant is born are provided by health visitors in all municipalities with the number and content depending on families’ specific needs. To avoid transmissions of the intervention to the control group, participants from the control group will receive visits from health nurses not trained in providing the COS-P program. Number of consultations with midwifes and visits from health nurses will be monitored for all participants.

### Recruitment

The referring midwifes from Herlev-Gentofte Hospital will recruit pregnant women who fulfills the inclusion criteria around 13–29 gestational weeks. Study inclusion can occur until after the second midwife consultation, if the midwife and health visitor arrange the joint consultation with the pregnant woman before gestational week 32. As part of the intervention, the midwife should screen the pregnant women for depressive and anxiety symptoms and risk of personality disorders before the joint consultation, but for late-included participants the screening can take place after the joint consultation. The women will be recruited to the project through an invitation and information leaflet sent from the hospital along with a notice of the first midwife consultation. Project personnel will then contact the women by phone to ascertain whether they are interested in participating, followed by a visit to potential participants in their homes to provide further information on the project, obtain informed consent, and enroll interested women and their families in the project. As part of the information about participation in the study the participants are informed that they can withdraw from the study at any given time if they wish so. We will ask those who decline the invitation to the study to identify their reasons for not participating.

**Fig. 1:**
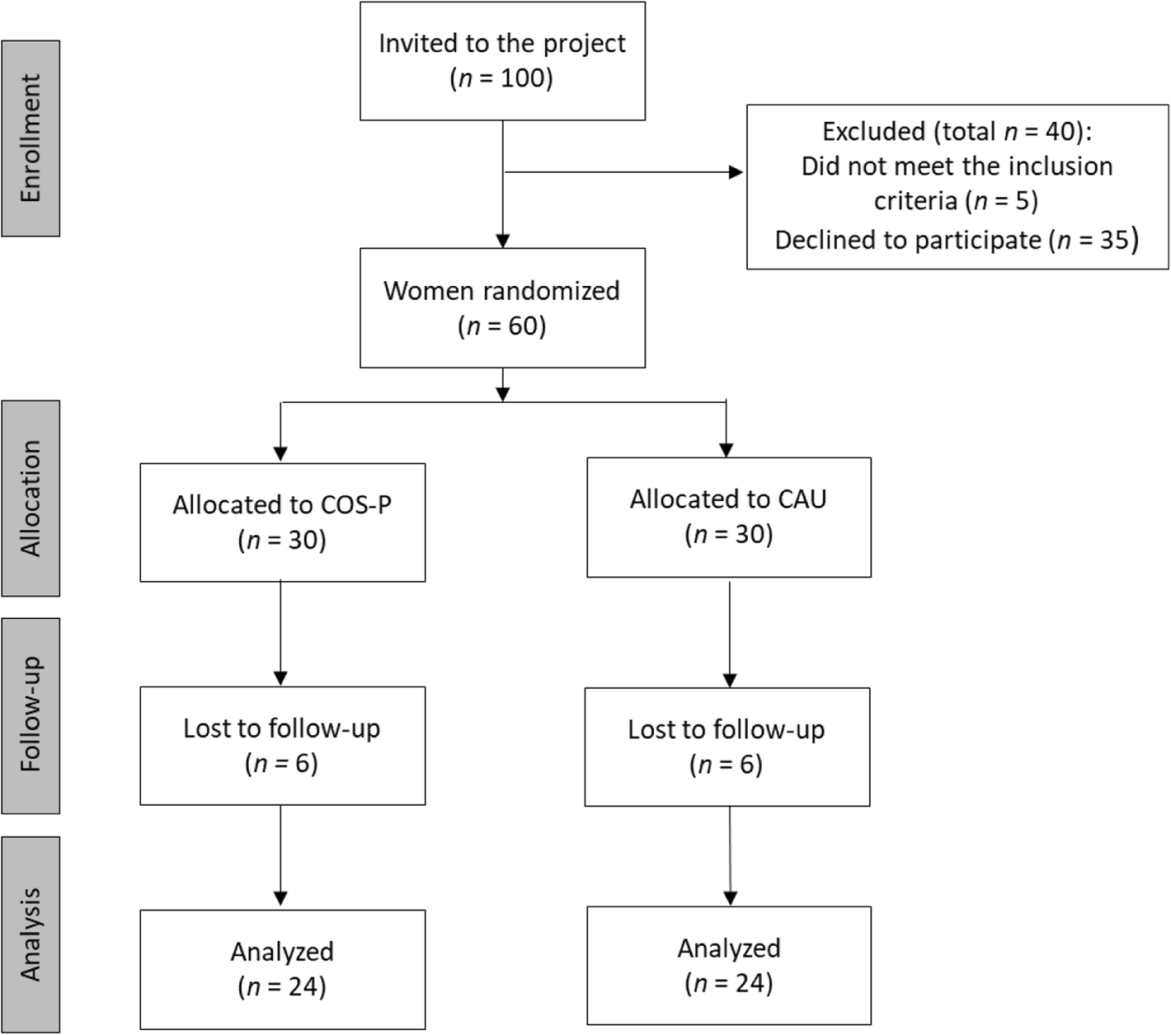
Planned recruitment process

In total, we expect that the project can identify 100 pregnant women from the target group from the four municipalities during the inclusion period of 10 months. An expected drop-out due to exclusion 40% and loss to follow-up 20% will result in an expected amount of 48 pregnant women who can complete the project. See Fig. 1.

### Randomization

The study population will be block randomized 1:1 to the intervention and control group, respectively. The randomization is stratified within each municipality. A research assistant will generate the allocation list. An individual who has no affiliation with the project will prepare the envelopes. Project personnel visiting the families will carry out the randomization (by opening the envelopes) after collecting the baseline data, and the family will be informed about which group they enter at the same visit.

### Procedure

Data will be collected at three points: at study inclusion at 3–5 months of pregnancy, when the infant is 8 weeks old, and after the intervention has ended when the child is 9 months old. Data will be collected via questionnaires delivered and answeredin the participant’s homes. Maternal sensitivity will be assessed at 8 weeks and 9 months based on video recordings of 5 mins of free mother-infant interactions also in the participants home. The control group will be assessed at the same follow-up periods as the intervention group. As an incentive to complete the questionnaires, participants will receive a gift with the value of approximately 300 Danish Crowns (USD 45) after completing the second follow-up questionnaire.

Different questionnaires will be developed for mothers and fathers/partners. The questionnaires will be similar, but the mother’s questionnaire will contain questions about her attachment to the child while pregnant (baseline), her attitude about and perceptions of breastfeeding (baseline) and how she feeds her baby (first and second follow-up).

The questionnaires will be pilot tested by individuals from the target group to ensure that they include a manageable number of items and that questions are understandable and relevant. The questionnaires will be revised according to the results of the pilot testing.

Participants in the intervention group who after enrollment do not wish to receive the intervention will be asked to stay in the study and complete the follow-up questionnaires if they wish so.

### Primary outcome

*Maternal sensitivity* will be measured by coding a five-minute mother-infant interaction sequence, using the “Coding Interactive Behavior Manual” (CIB) [62]. Sensitive response, or the ability to respond appropriately to the child’s attachment needs, is found to be the most reliable predictor of attachment security [14, 16]. The CIB has been validated in several studies across cultures and age groups [63].

Mother-infant interactions will be videotaped during a home visit by a researcher at the first follow-up when the child is 8 weeks old and at the second follow-up after the intervention has finished and the child is around 9 months old. A reliable coder, trained by Ruth Feldman, who is blind to treatment allocation, will code the mother-infant interactions. To reduce the risk of coder bias between the two time points, it will be ensured that the two time points will be coded with at least 6 months intermission. Inter-coder agreement will be assessed using a randomly selected subset of 20% of interactions coded by a second coder also blind to treatment allocation. Internal consistency of the original CIB-sensitivity composite will be calculated (Cronbach’s alpha). Only items contributing to the overall composite, defined as item-total correlations of .3 or higher, will be included in the study composite used in analyses [64].

### Secondary outcomes

Information on secondary outcomes will be collected from registers and self-report questionnaires. Table 2 summarizes the data collected from questionnaires at each assessment time. In addition, potential confounding variables such as infant Apgar scores and weight, length, and head circumference at birth will be obtained from registers.

**Table 2:** Measures

	Baseline Pregnancy, 2nd trimester	Follow-up 1 Infant aged 8 weeks	Follow-up 2 Infant aged 9 months
Maternal sensitivity (CIB)		X	X
Infant socio-emotional development (ASQ-SE)			X
Parental mentalization (P-PRFQ & PRFQ)	X	X	X
Parenting stress (PSI)			X
Parental depressive symptoms (EPDS)	X	X	X
Parental wellbeing (SWEMWBS)	X	X	X
Maternal and paternal attachment (ECR-S)	X	X	X
Maternal and paternal experience of social support (FSS)		X	X
Breastfeeding attitudes	X		
Breastfeeding status		X	X
Maternal attachment to the unborn child (MAAS)	X		
Self-reported previous and current mental illness	X		

*Infants’ socio-emotional development* will be measured with the Ages and Stages Questionnaire – Social Emotional (ASQ-SE). The mother and father/partner will independently complete the questionnaire at second follow-up with their responses assessed separately. The ASQ-SE examines self-regulation, compliance, communication, adaptive functioning, autonomy, emotions and interaction with other people [65].

*Parental reflective functioning* (PRF) is the capacity to focus on experience and feelings in one self and in the child. PRF after birth will be measured by the Parental Reflecting Functioning Questionnaire (the PRFQ [66];). Three subscales are used: (a) interest and curiosity in mental states, (b) the ability to recognize the opacity of mental states, and (c) non-mentalizing modes characteristic of parents with impairments in PRF (e.g, malevolent attributions, inability to enter the subjective world of the child) [66]. Validation studies of the PRFQ provide evidence for its reliability and validity. The version used in this study was translated by Mette Skovgaard Væver and Johanne Smith-Nielsen, Copenhagen University BabyLab.

*Parenting stress* will be measured using the Parenting Stress Index -short form (PSI-SF). The PSI-SF comprises three subscales—parental distress, parent-child dysfunctional interaction, and difficult child—as well as the total stress scale [67]. Studies provide psychometric support for the PSI-SF as an effective and appropriate measure for use with high-risk families [68].

*Parental depressive symptoms* will be assessed with the Edinburgh Postnatal Depression Scale (EPDS) at baseline and first and second follow-up visits. EPDS has high sensitivity and specificity for detecting clinical depression, using a clinical psychiatric diagnosis of depression as the reference [60].

*Parental wellbeing* will be measured using the Warwick Edinburg Mental Well-being Scale (WEMWBS), which reflects a broad understanding of well-being that includes affective-emotional aspects, cognitive-evaluative dimensions, and psychological functioning. All items are worded positively and address aspects of positive mental health. The 14-item WEMWBS shows high levels of internal consistency and reliability [69]. To reduce respondent burden, the current study will use the Short Warwick Edinburgh Mental Well-Being Scale (SWEMWBS), a seven-item version that is appropriate for monitoring mental well-being in populations [70]. Line Nielsen, Carsten Hinrichsen, Ziggi Ivan Santini and Vibeke Koushede at University of Southern Denmark translated the SWEMWBS for use in a Danish context [71].

The mothers’ attitude towards breastfeeding will be assessed at baseline and self-reported breastfeeding status will be assessed at first follow-up. At baseline, the mothers will be asked about their attitude towards breastfeeding, how they expect to feed or feed their baby and when they expect to stop or stopped breastfeeding.

### Background factors

Information on socioeconomic status, general wellbeing, family relations, social network, lifestyle, depressive symptoms, attachment style, maternal attitude towards breastfeeding and psychiatric and/or psychological treatment in the health care system in relation to mental illness will be gathered to gain insight into factors that can influence participants’ parenting skills and the outcomes of interest (Table 2).

*Adults’ attachment in close relationships* will be measured with the 12-item short form of the Experiences in Close Relationship Scale – Short Form (ECR-S) [72], which was developed from the 36-item Experiences in Close Relationship scale [73]. The items address anxiety and avoidance related to adult attachment. Attachment anxiety is defined as fear of interpersonal rejection or abandonment, an excessive need for approval from others and distress when the partner is unavailable. Attachment avoidance is defined as fear of dependence and intimacy and excessive need for self-reliance. People who score high on one or both dimensions are assumed to have an insecure adult attachment orientation [72]. Validity studies indicate that the ECR-S provides a reliable and valid measure of adult attachment [72]. Barbara Hoff Esbjørn, at University of Copenhagen translated the Danish version of the ECR used in this study.

*Parents’ perceived support from family, friends, society and their partner* will be measured with the Family and Social Support Scale (FSS) at the first and second follow-up visits, and both parents will be asked to complete it [74].

Maternal attachment to the unborn child will be measured at baseline with *the Maternal Antenatal Attachment Scale* (MAAS), which assesses thoughts, feelings and behavior towards the unborn child. The MAAS measures two dimensions of maternal attachment: quality (emotional closeness/distance, positive/negative feelings, tenderness/irritation aimed at the unborn child) and intensity/the amount of time the mother is in attachment mode, i.e., how involved the pregnant woman is with her fetus [75].

PRF during pregnancy will be measured using *the Prenatal Parental Reflective Functioning Questionnaire* (P-PRFQ) [76]. The P-PRFQ has been adapted from the Parental Reflective Functioning Questionnaire (PRFQ) [66] to include items specific to the pregnancy phase and is feasible for use with both mothers and fathers [76]. The study investigators translated the P-PRFQ into Danish for use in the current study with permission from its originators.

### Qualitative interviews

The joint consultation with the midwife and the health visitor and the pregnant woman will be evaluated using semi-structured qualitative interviews. Further, the semi-structured interviews will be used to evaluate the midwives’ experiences with the screening tool and the health nurses’ experiences with providing COS-P to the families. All the participating health nurses and midwifes will be interviewed. The interviews with the health nurses will be conducted as focus group interviews. The interviews with the midwives will be individual interviews. Further, around 30 semi-structured interviews will be conducted with the participating parents. The interviews will be conducted at first follow-up and second follow-up. The interviews will focus on the parents’ experiences of the care they have received during their pregnancy and postnatal period. Parents from the intervention group will specifically be asked about how they experienced being asked questions about depression, anxiety and their personality, the joint consultation between their midwife and their health nurse and receiving COS-P.

### Statistical methods

#### Sample size

The primary outcome of maternal sensitivity will be assessed with the Coding Interactive Behaviour Manual [62], which uses an interval scale from 1 to 5, with low numbers indicating low sensitivity. For the power analyses, it was assumed that the average score at baseline would be 3 with a standard deviation of 0.9. This is based on a literature review regarding the Coding Interactive Behaviour Manual [77]. Mothers will be video filmed in interaction with their infant at the first and second follow-up visits. A study examining maternal sensitivity in postpartum depressed mothers found a difference of 0.9 between depressed and nondepressed mothers [78]. Consequently, we aim to detect a minimal difference of 0.75 between groups. Based on a t-test with α of 0.05, we should include 24 mothers in each group to obtain a power on 80%. A likely dropout rate of 20% yields a sample size of 60 (*n* = 30 in each group) at randomization. The recruitment process will continue until at least 60 vulnerable pregnant women are included in the study.

#### Compliance

High compliance with the intervention is defined as completion of seven out of the nine training sessions from COS-P.

#### Data management

Data management will comply with the rules of the Danish Data Protection Agency. All study data will be managed using REDCap electronic data capture tools [79], double data entry, range checked for data values, checked against the paper-based assessments and exported to SAS Enterprise Guide 7.1 (SAS Institute Inc., Cary, NC, USA).

#### Access to data

Researchers from Center for Clinical Research and Prevention involved in the study (AKA, JP, KR and MS) as well as a statistician from Center for Clinical Research and Prevention will have access to the final trial dataset.

#### Statistical analyses

All analyses will be blinded and performed using SAS Enterprise Guide 6.4. Analysis and presentation of data will be in accordance with the CONSORT guidelines [80]. Descriptive data for the intervention and control groups will be compared using the chi-square test for categorical variables, the Student’s t test for normally distributed continuous variables, and the Mann-Whitney U test for non-parametric variables. Descriptive data will be presented as means with standard deviations, medians with inter-quartile ranges or frequencies with percentages depending on the distribution of the variable.

An ANOVA analysis of the primary outcome maternal sensitivity on randomization group will be performed. The primary outcome will be the between-group difference at second follow-up. The primary analysis will follow the intention-to-treat principle using multiple imputation in case of missing outcome measures and will be unadjusted. Secondary, this analysis will be adjusted for background variables with a shew distributions between intervention and control group, and the analysis will be analyzed using the per protocol principle where only participants with a high compliance will be in the intervention group. To assess whether an effect of the intervention is diluted by the inclusion of non-completers (participants from the intervention group participating in four or fewer sessions of COS-P) the analyses described above will be repeated excluding non-completers from the intervention group. Similar analysis will be performed on secondary outcomes at the second follow-up. For secondary analysis where baseline values are measured an ANCOVA model on development in outcome from baseline to follow-up adjusted for baseline values will be used.

The additional aim of identifying psychological risk factors important for prenatal bonding will be explored by performing a series of multiple regression with maternal-fetal relationship as the primary outcome. Adult attachment style, depressive symptoms, and prenatal parental reflective functioning are the independent variables. The dependent variable of maternal-fetal relationship will be analyzed in two separate regression models predicting “the intensity of involvement with the fetus’ and ‘the quality of the maternal-fetal relationship” as it has been suggested to distinguish between the two sub-scales (van Bussel et al., 2010). We will control for the effect of gestational age and parity, maternal education and age.

The final aim of identifying elements important for the implementation process will be answered by performing qualitative analysis of interviews with professionals and the participating families.

All models will be investigated for goodness-of-fit (linearity, variance homogeneity and normal distribution of residuals) by visual inspection of plots and remodeling will be performed accordingly. All between-group differences will be expressed as the average difference for non-transformed data. If log transformation is necessary for any outcome, results will be shown as percentage difference. *P* values ≤0.05 will be considered statistically significant. However, for all analyses evaluating potential modifiers and confounders of the intervention *p* values ≤0.01 will be used to account for multiple testing. No interim analysis will be made.

### Ethical considerations

The project was reported to The Committee on Health Research Ethics of the Capital Region of Denmark with protocol number 17006186. The project was assessed as not registrable and can be implemented without further permission.

### Scientific dissemination

Three articles on the results of the project are planned for publication in international peer-reviewed journals: 1) One article reporting on prenatal maternal risk profiles, 2) one RCT-article reporting on the effect of the intervention on both primary and secondary outcomes, and 3) one article focusing on elements of importance for implementation of the intervention reporting on data from the qualitative interviews with midwifes, health nurses, and parents regarding their experiences with the perinatal care model, they delivered or were allocated to.

## Discussion

The protocol describes the design and evaluation of an early intervention program aiming to enhance maternal sensitivity in psychosocially vulnerable families. If proven effective, the intervention will make available an advantageous approach to improving mental health for vulnerable families. The evaluation will also provide new information on prenatal screening of at-risk families, initiation of the parenting intervention COS-P as provided by trained health visitors as individual parental education at home and during pregnancy and evaluate a perinatal collaboration between midwifes and health nurses. Similar evaluations have not yet been reported in Denmark or elsewhere.

## Data Availability

Not applicable in this article as the study is ongoing and all data not yet collected.

## Abbreviations

ASQ-SE: Ages and Stages Questionnaire – Social Emotional (ASQ-SE)
CIB: Coding Interactive Behavior
COS: Circle of Security
COS-P: Circle of Security Parenting
ECR-S: Experiences in Close Relationship scale
EPDS: Edinburgh Postnatal Depression Scale
FSS: Family and Social Support Scale
MAAS: Maternal Antenatal Attachment Scale
PSI: Parenting Stress Index
PSI-SF: PSI Short Form
PRF: Prenatal reflective functioning
P-PRFQ: Prenatal Parental Reflective Functioning Questionnaire
PRFQ: Parental Reflective Functioning Questionnaire
SAPAS: Standardized Assessment of Personality
WEMWBS: Warwick Edinburg Mental Well-being Scale
